# Protocol for a prospective study evaluating circulating tumour cells status to predict radical prostatectomy treatment failure in localised prostate cancer patients (C-ProMeta-1)

**DOI:** 10.1186/s12885-023-11081-0

**Published:** 2023-06-23

**Authors:** Tarek Al-Hammouri, Ricardo Almeida-Magana, Rachel Lawrence, Tom Duffy, Laura White, Edwina Burke, Sakunthala Kudahetti, Justin Collins, Prabhakar Rajan, Daniel Berney, Rhian Gabe, Greg Shaw, Yong-Jie Lu

**Affiliations:** 1 Dept. of Urology, University College London Hospitals, London, UK; 2 Queen Mary University of London, Barts Cancer Institute, London, UK; 3 Division of Surgery and Interventional Science, University College London, London, UK; 4 Queen Mary University of London, Wolfson Institute of Population Health, London, UK

**Keywords:** Prostate cancer, Radical prostatectomy, Micro-metastasis, Circulating tumour cells

## Abstract

**Background:**

Treatment decisions in prostate cancer (PCa) rely on disease stratification between localised and metastatic stages, but current imaging staging technologies are not sensitive to micro-metastatic disease. Circulating tumour cells (CTCs) status is a promising tool in this regard. The Parsortix® CTC isolation system employs an epitope-independent approach based on cell size and deformability to increase the capture rate of CTCs. Here, we present a protocol for prospective evaluation of this method to predict post radical prostatectomy (RP) PCa cancer recurrence.

**Methods:**

We plan to recruit 294 patients diagnosed with unfavourable intermediate, to high and very high-risk localised PCa. Exclusion criteria include synchronous cancer diagnosis or prior PCa treatment, including hormone therapy. RP is performed according to the standard of care. Two blood samples (20 ml) are collected before and again 3-months after RP. The clinical team are blinded to CTC results and the laboratory researchers are blinded to clinical information. Treatment failure is defined as a PSA ≥ 0.2 mg/ml, start of salvage treatment or imaging-proven metastatic lesions. The CTC analysis entails enumeration and RNA analysis of gene expression in captured CTCs. The primary outcome is the accuracy of CTC status to predict post-RP treatment failure at 4.5 years. Observed sensitivity, positive and negative predictive values will be reported. Specificity will be presented over time.

**Discussion:**

CTC status may reflect the true potential for PCa metastasis and may predict clinical outcomes better than the current PCa progression risk grading systems. Therefore establishing a robust biomarker for predicting treatment failure in localized high-risk PCa would significantly enhance guidance in treatment decision-making, optimizing cure rates while minimizing unnecessary harm from overtreatment.

**Trial registration:**

ISRCTN17332543.

## Introduction

Prostate cancer (PCa) is the most commonly diagnosed non-cutaneous cancer in Western male patients. Although most cases are indolent and have a low risk of progression, it remains a significant cause of mortality, with over 375,000 deaths recorded worldwide in 2020 [1]. The presence of metastasis is the most important risk factor for PCa-specific mortality [2] and patients are currently deemed to have localised PCa when staging evaluations based on Computed Tomography (CT) and 99mTc whole-body Bone Scans (BS) are negative for metastasis [3, 4]. However, the sensitivity of both techniques is low, and although this can be improved using molecular-based imaging such as Prostate Specific Membrane Antigen (PSMA) Positron Emission Tomography (PET), small volume metastasis or micro-metastasis can still be missed [5, 6].

In its localised stage, clinically significant PCa is amenable to cure by treatments such as radical prostatectomy (RP), radical radiotherapy (RT), brachytherapy, or ablative focal treatments [7], whereas the metastatic stage is best treated with systemic therapies such as androgen deprivation therapy (ADT) ± chemotherapy [4]. Nonetheless, 40% of high-risk cases treated with RP recur within 2 years, indicating the presence of minimal residual disease (MRD) following surgery, potentially due to pre-surgery micro-metastasis in the form of circulating tumour cells (CTCs). These patients require additional treatment and are at a high risk of PCa-specific mortality [8].

The existence of CTCs was first identified in the nineteenth century [9]. Owing to the challenges associated with identifying and/or separating CTCs from the rest of the blood components, it was not until 2008, that the Food and Drug Administration (FDA) approved the first test (CellSearch) for detecting CTCs in blood samples for the prognosis of several types of cancers at the advanced stage [10]. However, the CellSearch platform isolates CTCs based on EpCAM expression and will therefore miss CTCs that have undergone epithelial-to-mesenchymal transition (EMT). This limitation explains why some studies have failed to correlate CTCs with oncological outcomes in prostate cancer [11].

The epitope-independent Parsortix® CTC isolation system relies on cell size and deformability to detect CTCs, allowing isolation of CTCs across the EMT spectrum [12]. This system has recently gained FDA approval for CTC isolation in breast cancer [12]. In our previous study comparing metastatic and localised PCa, CTC count was significantly associated with metastatic cancer [13]. We detected CTCs in all patients with metastatic PCa but also in more than half of the localised PCa cases. Another study by our group, using pre-biopsy patient blood samples, found that the combination of CTC score, prostate-specific antigen (PSA) level, and CTC gene expression gave an AUC of 0.927 for the prediction of clinically significant PCa post-biopsy [14]. Nevertheless, the potential of this technology in predicting the oncological outcome of localized PCa has yet to be evaluated.

We hypothesise that detection of CTCs by the Parsortix® system is an accurate indicator of micro-metastasis and in combination with CTC gene expression may predict future metastasis development. This study aims to investigate the accuracy of CTC status in predicting cancer recurrence after radical prostatectomy in a cohort of patients diagnosed with unfavourable intermediate, high- or very-high risk localised prostate cancer.

## Objectives

The primary objective is to determine the accuracy of pre-operative CTC positivity in predicting post-RP treatment failure as defined by biochemical recurrence (BCR) or new metastatic lesions. The co-primary objective is to establish the accuracy of a combined CTC test that incorporates both CTC enumeration and gene expression, in order to predict post-RP treatment failure.

The secondary objectives are:

1. Investigating the potential of pre-surgery CTCs to predict positive lymph node involvement. Positive lymph node involvement is a crucial factor that influences the selection and scale of RP as the initial treatment.
2. Provide a preliminary assessment of the prognostic value of pre- and post-surgery CTC data, specifically CTC positivity and gene expression, in predicting the development of post-RP prostate cancer (PCa) metastasis, overall survival, and cancer-specific survival.
3. To generate evidence and data to design a future clinical trial centred around a CTC-based intervention to guide therapeutic choice in the management of high-risk localised PCa.

### Trial design

**Fig. 1:**
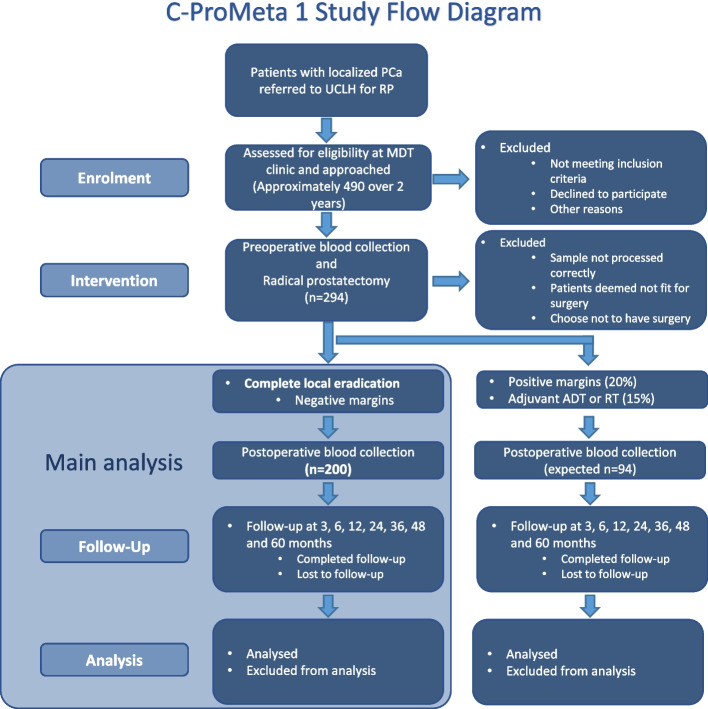
CONSORT diagram – C-ProMETA-1 study. *PCa* Prostate Cancer, *RP* Radical Prostatectomy, *MDT* Multi-disciplinary Meeting, *ADT* Androgen deprivation therapy, *RT* Radiotherapy

Observational single-site, double-blinded, prospective, cohort study. This report follows the SPIRIT guidelines [15]. See CONSORT diagram (Fig. 1). This study is registered on ClinicalTrials.gov with the number NCT05533515.

## Methods

### Study population and study setting

Patients are referred to University College London Hospitals (UCLH) trust for counselling regarding PCa treatment options. If surgery is their preferred choice and they meet the eligibility criteria, patients are invited to enrol in the study at their pre-surgical consultation. Study procedures and outcomes are explained and patients will receive the study patient information sheet. Patients who agree to take part will sign the approved consent form. All patients who fulfil the inclusion criteria are invited to participate. Diagnosis of non-metastatic disease is based on the current standard diagnostic imaging methods including CT scans, multi-parametric magnetic resonance imaging (mpMRI), Bone Scan (BS) and/or PSMA-PET scan.

### Eligibility criteria

#### Inclusion criteria:

1. Patients with unfavourable intermediate, high- or very-high risk non-metastatic PCa based on the National Comprehensive Cancer Network (NCCN) guidelines.
2. Scheduled for robot-assisted RP at UCLH.
3. Patients who sign the informed consent form.

#### Exclusion criteria:

1. Diagnosis of another synchronous cancer.
2. Patients who had received previous PCa treatment.
3. Patients who receive neo-adjuvant ADT.

#### Withdrawal criteria:

1. Enrolled patients deemed unfit or unwilling to undergo RP.
2. Failure to process the pre-op sample (technical issues).
3. Withdrawal of consent to continue participating in the study.

### Interventions

#### Radical prostatectomy

After PCa diagnosis and staging, RP is performed using a minimally invasive approach with robot-assisted surgery as the standard of care in the National Health Service (NHS). RP involves the complete removal of the prostate and seminal vesicles with or without lymphadenectomy. Tissue-sparing approaches are guided by a pre-surgical mpMRI-based planning meeting.

### CTC analysis

Twenty millilitres of peripheral blood is collected from each patient during the pre-and post-RP PSA test blood acquisition. Samples are taken to the laboratory at the Barts Cancer Institute (BCI), John Vane Science Centre, Charterhouse Square. We aim to process samples within 4 h of acquisition and the procedure is carried out as described previously [12]. Briefly, the buffy-coat is isolated from 7 mL whole blood, washed with phosphate buffered saline (PBS), and diluted in isolation buffer (PBS containing 1% bovine serum albumin and 2 mM ethylenediaminetetraacetic acid (EDTA)). The cell solution is added back to the original vacutainer containing 0.5 mL whole blood and placed into the Parsortix® system. The sample is passed through the Parsortix® microfluidics to a cassette where the cells are separated based on size and deformability. Harvested cells including CTCs are fixed with acetone on a microscope slide. Immunofluorescence analysis involves staining the slides for antibodies that target the leukocyte-specific marker CD45-Allophycocyanin (Miltenyi biotec cat no. 130–113-114), the epithelial marker Cytokeratin-Fluorescein Isothiocyanate (Miltenyi biotec cat no. 130–118-964), and the mesenchymal marker Vimentin-AlexaFluor 568 (abcam cat no. ab202504). Cellular DNA is identified using 4’,6-diamidino-2-phenylindole (DAPI). CTC slides are scanned on a Nanozoomer S60 fluorescence microscope (Hamatsu®) using a 20 × objective. For the CTC RNA extraction, CTCs are harvested from the Parsortix® and added to 2 × lysis buffer solution (Angle) in 1:1 ratio. The vial is vortexed and stored at -80°C prior to analysis. Remaining blood samples are separated into plasma and peripheral blood mononuclear cells and stored at -80°C in the Robert Lane Tissue Bank (RLTB) for further research use.

### Participant timeline

Blood samples are taken for CTC analysis before surgery during routine pre-surgery testing. At routine 3-month post-surgery follow-up PSA testing, patients are invited to donate a second blood sample if they have not undergone additional treatments such as hormonal or radiation therapy. Clinical follow-up is carried out at 3, 6- and 12-months post-op and then on an annual basis. Medical records will be reviewed until the end of the project to ascertain whether subsequent PSA tests are positive, indicating BCR (PSA ≥ 0.2 ng/mL), imaging-proven metastasis, or if they have received salvage treatment with hormones/radiotherapy, PCa-specific mortality or other causes. Table 1 summarizes study assessments.

**Table 1:** Patient recruitment, sample collection and follow-up procedure

Procedure	Screening/ Baseline	Pre-RP	3 months post-op	6 months post-op	12 months post-op	Annual follow-up 2 to 10 years
Eligibility assessment	x					
Informed Consent	x					
Blood Sample collection		x	x			
PSA		x	x	x	x	x
Medical record review and online app/phone follow-up; (PSA and/or Adjuvant therapies) rates of BCR, metastasis, other progression events and death				x	x	x

*RP* Radical Prostatectomy, *BCR* Biochemical recurrence. *PSA* Prostate specific antigen

### Outcome measures

#### Primary outcome

The primary outcome is post-RP treatment failure. The primary analysis will occur after 4.5 years of follow up after the start of recruitment. Post-RP treatment failure is defined as a PSA ≥ 0.2 ng/mL at routine PSA testing after RP. If patients have a rising PSA trajectory and clinicians recommend additional treatment before reaching the 0.2 ng/mL threshold, this will also be considered a treatment failure. Additionally, any metastatic lesion detected by imaging tests independent of the PSA level will be considered a treatment failure. This combined post-RP treatment failure primary endpoint will maximise capture of all clinically significant cancer recurrence events.

### Secondary outcomes

1. BCR during the first 4.5 years of follow-up: PSA ≥ 0.2 ng/mL at any time post-RP and remaining at this level or further increase afterwards without further treatment.
2. Metastasis (any location)-free survival during the first 4.5 years of follow-up.
3. Deaths from any cause during the first 4.5 years of follow-up.
4. Prostate cancer-specific deaths during the first 4.5 years of follow-up.
5. Metastasis (any location)-free survival at 10 years follow-up.
6. Prostate cancer-specific survival at 10 years of follow-up.
7. Overall survival at 10 years of follow-up.

### Criteria for discontinuing or modifying allocated interventions

Patients with positive surgical margins (PSM) or positive lymph nodes based on histopathological examination will be analysed separately. Patients can withdraw their consent at any time by sending an email or letter with their contact details to the address provided in their patient information sheet (PIS). If the donated samples have not already been used for research purposes, they will be removed from storage and destroyed, as well as any data that may have been collected and stored.

### Relevant concomitant care permitted or prohibited during the trial and provisions for post-trial care

Patients are excluded from the trial analysis if they receive ADT before surgery as this may interfere with the accuracy of the CTC status. However, treatment decisions are not informed by the CTC results. All clinical staff and patients are blinded to CTC results. Therefore, standard of care treatment interventions will be decided by clinical care staff who are blinded to patient CTC status. Follow-up at 3 months is done at UCLH, subsequent follow up is done at their corresponding local hospitals or General Practitioner (GP) practice.

### Blinding

To avoid influencing standard patient treatment, management, and progression outcomes after RP, the patients and all members of the clinical care team are blinded to the CTC results. Only the laboratory team is aware of the patients’ CTC status. The laboratory team are blinded to the patients’ clinical status, progression, and outcomes. There are no criteria for unblinding, as this is an observational study. The study findings will not affect patient care for the participants during the course or after the end of the study period.

### Data management

Each patient is allocated a unique pseudo-anonymised ID used by the laboratory research team. This unique ID is recorded in the RLTB database for future correlation analysis of CTC results with clinical follow-up data. There will be fewer patients for post-RP than for pre-RP blood collection based on patient exclusion criteria after RP. Completed consent forms are handed over to the RLTB for secure storage.

The data generated from this study includes immunofluorescence images of CTCs, RNA expression data of metastasis-associated genes in CTCs, and follow-up data on clinical and cancer progression associated with PCa patients. These data are also securely stored in a designated folder on the BCI IT server. The data is curated throughout its life cycle (during the study and before the data is published). Each digital PCR is performed using Angle® optimized method for multiplex CTC gene expression analysis. Gene expression value is recorded in the original data format and saved in the designated folder in the BCI server and will be available for data sharing after publication.

### Statistical methods

In the reporting of this study, we will adhere to the STARD guidance (ref: https://www.equator-network.org/reporting-guidelines/stard/). The study flow and patient characteristics will be reported. Accuracy measures will be presented along with 95% confidence intervals (CIs). The primary analysis will focus on estimating the accuracy of pre-surgery CTC score positivity in detecting post-RP treatment failure, as defined above. We will report the observed specificity, positive predictive values, and negative predictive values. Furthermore, specificity will be presented over time. To address the potential artificial inflation of false positives due to increasing prevalence over time, additional analyses will be conducted. These analyses will be repeated for the CTC test, which combines CTC score positivity with CTC gene-expression analysis.

Recurrence events and follow-up CTC results will be reported and compared over time. BCR-free survival will be estimated, and Kaplan–Meier curves will be presented and stratified by CTC test result. Likewise, metastases-free, prostate cancer and overall survival will be reported and stratified by CTC test result. CTC data will be explored along with other pre-operative risk factors using multivariate analysis. No interim analysis is planned. A subgroup analysis will be performed to analyse the predictive value of pre-surgery CTCs in patients with positive surgical margins (PSM), lymph node metastasis, or those who received adjuvant treatment.

### Consideration with respect to missing data

The primary analysis is concerned with measures of accuracy and only data which has known positive or negative values will be included. The study flow diagram will aid interpretation and show the number of participants recruited who did not provide study samples. The number of samples collected, analysed and providing data contributing to these accuracy measures will be reported.

Kaplan–Meier curves will be reported with the numbers at risk over time. All eligible participants providing valid, blood samples which are analysable using the CTC tests will be included in analyses. Time to event will be from date the blood sample was taken up to the point an event occurs, or death, loss to follow up, withdrawal, or the analysis cut-off date, whichever occurs first. Data from those not experiencing an event will be right censored. Reasons for loss to follow up or withdrawal will be collected where possible and monitored by the TSC. Non-informative censoring will be used in this study where the patient and clinical team are blinded from the CTC test result and where the CTC result is not used to influence care and therefore drop out.

Multivariable regression/analyses will be on a complete case basis, with numbers included in the analysis report.

### Sample size

Consistent with previous work, this study estimates that CTCs can predict post-RP cancer events with 95% sensitivity. During the first 4.5 years of follow-up, the hypothesised prevalence of BCR events in high-risk PCa is at least 40%. To confirm an expected sensitivity of 95% within 5% precision on either side of a 95% confidence interval (CI) in a sample with an underlying prevalence of 40% requires at least 73 events, that is at least 183 recruited individuals. We inflated this by 8.5% to target a recruitment of 200 participants to ensure the suitability of samples and measurable outcomes.

We expect to screen approximately 490 patients over 2 years and recruit 294 patients to compensate for the 20% PSM and 15% adjuvant therapy rates in patients undergoing surgery. This will allow us to evaluate 200 paired pre- and post-surgery CTC samples. Specificity is important considering the future circumstance of potentially denying curative surgery if a CTC test falsely indicates metastasis. A study size of 200, including 120 true negatives, would provide a 97.5% lower confidence limit with a specificity of 99% (1% false positive rate). However, we anticipate that the specificity will be lower due to positive results for those who experience recurrence beyond the 5-year study period. Assuming that these events are due to micro-metastasis at the time of surgery, observed sensitivity and specificity will be used, plus additional analyses assuming that up to 20% of the events occur after the funded 5-year project to estimate the real false positive and negative rates.

### Plans to give access to the full protocol, participant level data and statistical code

When the relevant safeguards have been applied for intellectual property and we have published the main findings, anonymised data of the findings will be publicly accessible. The research data on the main outcomes will be made available as a spreadsheet through a web link published in scientific journals and/or the BCI Institute’s website.

### Oversight and monitoring

The study management group (SMG), consisting of the PIs/Co-Is, key collaborators, lab team members and statistician convenes every month. The Trial Steering Committee (TSC), which includes a patient member, a consultant urologist (chair), a biomarker study specialist/statistician, and a basic research scientist independent from the study, monitors recruitment and study progress in terms of outcome data collection and statistical assumptions underpinning the study, including a confidential review of the event rates. The TSC meets on an annual basis. Due to the observational nature of the study, there is not a separate data monitoring committee.

### Adverse event reporting and harms

Obtaining blood samples for this study includes the risks associated with venepuncture, such as temporary discomfort and bruising. Blood samples for pre-operative preparation and pre-and post-operative PSA monitoring are part of the NHS standard of care for PCa patients; therefore, obtaining samples for this study does not involve any additional risk or burden for patients.

### Frequency and plans for auditing trial conduct

The Sponsor, funding body, and/or regulatory bodies may audit the study site or central facility. The PI will ensure an adequate quality and number of monitoring activities conducted by the study team. This will include adherence to the protocol, consenting procedures, and source data verification to ensure adequate data quality. The relevant PIs will inform the sponsor (Queen Mary University of London) should they have concerns which have arisen from monitoring activities, and/or if there are problems with any oversight or monitoring procedures. RLTB will be auditing consent forms on receipt, and study auditing will be carried out as per the local RLTB policy with prior agreement with the study team.

## Discussion

CTCs can be identified at an early stage of cancer development. They have been detected in certain cases at the breast cancer precursor stage, carcinoma in situ [16], and the presence of CTCs has also been shown to precede lung cancer diagnosis by a few years [17]. In 2017, the presence of CTCs in non-metastatic breast cancer cases was included in the TNM staging system and defined as cM0(i +) [11]. This has led to several trials investigating the potential of CTCs as a diagnostic and/or prognostic biomarker for PCa management. However, current available evidence is conflicting regarding the accuracy of CTC analysis for this purpose. In the castration resistant metastatic setting, CTC presence has been correlated with the risk of cancer progression [18]. Using the CellSearch system, Meyer et al. [19] evaluated a cohort of 152 patients treated with RP and found no correlation with the development of BCR. However, CTCs were only found in 11% of the cohort, this was probably due to the potential of missing CTCs in a blood sample of limited volume. Moreover, CTCs can also undergo possible mesenchymal transition, lowering their capture rate [20].

We have demonstrated that the Parsortix® system may have the potential to overcome these limitations [12]. In a previous study using this technology, we evaluated blood from patients with suspected PCa before their diagnostic biopsy. We found that CTCs were positive in 77% of clinically significant PCa cases and only 13% in latent or biopsy-negative PCa patients (based on post-biopsy classification). By comparing CTC number, CTC gene expression and PSA, we achieved a high accuracy (93%) in predicting the subsequent biopsy outcome of aggressive PCa [14]. Therefore, CTC status alone reflects the true potential for PCa metastasis and may predict clinical outcomes better than the current PCa progression risk grading systems [13].

Furthermore, following an extensive literature review and experimental validation, we identified 30 PCa prognostic genes specifically expressed in PCa CTCs (detectable using Fluidigm qRT-PCR in Parsortix® harvested cancer cells but not in normal control blood samples). By analysing these genes, we identified a 12-gene panel to distinguish clinically significant PCa with unfavourable characteristics from favourable disease [14]. In addition, a recent study in our group revealed that CTC number and CTC gene expression in combination may be predictive of treatment response in a cohort of PCa patients undergoing docetaxel chemotherapy [21]. By combining both CTC detection and gene expression analysis, we expect this study to provide an even better level of association that predicts cancer recurrence after surgery.

### Expected limitations

This is a single-site trial which could limit the external validity of the findings. However, our centre receives patients from several regional hospitals with a large catchment area with high ethnic diversity. Robot-assisted RP and clinical care procedures will be performed according to the current NHS standards of care. We expect the trial participants to be representative of a wider high-risk patient population. Another possible limitation of this study is the relatively short follow-up time of 4.5 years which limits the power to detect and observe differences, especially for overall survival and PCa-specific survival. Nonetheless, most cancer recurrences for high-risk PCa occur within the first five years of follow-up [22]. Depending on the availability of funding, our objective is to extend the follow-up period to 10 years or longer, allowing us to gather outcomes through NHS digital and other national registries, provided that patients give their consent for this extended monitoring.

### Ethical aspects

This study is conducted within the framework of the BCI, RLTB activity, with approval from the NHS Research Ethics Committee (REC) and with the Integrated Research Approval System (IRAS) Research Tissue Bank project ID 140998 ‘Collection of Genito-urinary tissue’. The current protocol is version 2.0. The Research Tissue Bank has obtained NHS REC approval, which is renewed every five years. The current REC approval is granted by the London—City & East Research Ethics Committee (reference number 19/L0/0994), and the next renewal date is scheduled for 15/07/2024. Written informed consent will be obtained from all patients. All methods will be carried out in accordance with relevant guidelines and regulations.

## Abbreviations

ADT: Androgen Deprivation Therapy
AUC: Area under the ROC Curve
BCI: Barts Cancer Institute
BCR: Biochemical Recurrence
BS: Bone Scans
CTC: Circulating Tumour Cell
DAPI: 4’,6-Diamidino-2-phenylindole
EDTA: Ethylenediamine Tetra acetic Acid
EMT: Epithelial to Mesenchymal Transition
MRD: Minimal Residual Disease
NHS: National Health Service
PBS: Phosphate Buffered Saline
PCa: Prostate Cancer
PI: Principal Investigator
PIS: Patient Information Sheet
PSM: Positive surgery margin
PSMA-PET: Prostate Specific Membrane Antigen – Positron Emission Tomography
QMUL: Queen Mary University of London
ROC: Receiver Operating Curve
RP: Radical Prostatectomy
RLTB: Robert Lane Tissue Bank
UCLH: University College London Hospital

## References

[1] Sung H, Ferlay J, Siegel RL, Laversanne M, Soerjomataram I, Jemal A (2021). Global Cancer Statistics 2020: GLOBOCAN Estimates of Incidence and Mortality Worldwide for 36 Cancers in 185 Countries. CA Cancer J Clin.

[2] Rider JR, Sandin F, Andrén O, Wiklund P, Hugosson J, Stattin P (2013). Long-term Outcomes Among Noncuratively Treated Men According to Prostate Cancer Risk Category in a Nationwide. Population-based Study Eur Urol.

[3] N. Mottet PC, R.C.N. van den Bergh, E. Briers, M. De Santis, S. Gillessen, J. Grummet, A.M. Henry, T.H. van der Kwast, T.B. Lam, M.D. Mason, S. O’Hanlon, D.E. Oprea-Lager, G. Ploussard, H.G. van der Poel, O. Rouvière, I.G. Schoots. D, Tilki, T. Wiegel, T. Van den Broeck, M. Cumberbatch, A. Farolfi, N. Fossati, G. Gandaglia, N. Grivas, M. Lardas, M. Liew, E. Linares Espinós, L. Moris, P-P.M. Willemse. EAU-EANM-ESTRO-ESUR-ISUP-SIOG Guidelines on Prostate Cancer: European Association of Urology; 2022. Available from: https://uroweb.org/guidelines/prostate-cancer. [cited 2023 May 17].

[4] Risko R, Merdan S, Womble PR, Barnett C, Ye Z, Linsell SM (2014). Clinical predictors and recommendations for staging CT scan among men with prostate cancer. Urology.

[5] 68Ga-PSMA PET/CT for Primary Lymph Node and Distant Metastasis NM Staging of High-Risk Prostate Cancer | Journal of Nuclear Medicine. Available from: https://jnm.snmjournals.org/content/62/2/214.long. [cited 2023 May 15]. 10.2967/jnumed.120.245605 32444374

[6] Gandaglia G, Mazzone E, Stabile A, Pellegrino A, Cucchiara V, Barletta F (2022). Prostate-specific membrane antigen Radioguided Surgery to Detect Nodal Metastases in Primary Prostate Cancer Patients Undergoing Robot-assisted Radical Prostatectomy and Extended Pelvic Lymph Node Dissection: Results of a Planned Interim Analysis of a Prospective Phase 2 Study. Eur Urol.

[7] Wilt TJ, Ullman KE, Linskens EJ, MacDonald R, Brasure M, Ester E (2021). Therapies for Clinically Localized Prostate Cancer: A Comparative Effectiveness Review. J Urol.

[8] Prognostic Value of Biochemical Recurrence Following Treatment with Curative Intent for Prostate Cancer: A Systematic Review - ScienceDirect. Available from: https://www.sciencedirect.com/science/article/pii/S0302283818307528?via%3Dihub. [cited 2023 May 15]. 10.1016/j.eururo.2018.10.011 30342843

[9] Ashworth TR. A case of Cancer in which cells similar to those in the Tumours were seen in the blood after death. The Australian Medical Journal. 1869:146–7.

[10] Allard WJ, Matera J, Miller MC, Repollet M, Connelly MC, Rao C (2004). Tumor Cells Circulate in the Peripheral Blood of All Major Carcinomas but not in Healthy Subjects or Patients With Nonmalignant Diseases. Clin Cancer Res.

[11] TNM Classification of Malignant Tumours, 8th Edition | Wiley. Wiley.com. Available from: https://www.wiley.com/en-gb/TNM+Classification+of+Malignant+Tumours%2C+8th+Edition-p-9781119263579. [cited 2023 May 17].

[12] Xu L, Mao X, Imrali A, Syed F, Mutsvangwa K, Berney D (2015). Optimization and Evaluation of a Novel Size Based Circulating Tumor Cell Isolation System. PLoS ONE.

[13] Xu L, Mao X, Guo T, Chan PY, Shaw G, Hines J (2017). The Novel Association of Circulating Tumor Cells and Circulating Megakaryocytes with Prostate Cancer Prognosis. Clin Cancer Res.

[14] Xu L, Mao X, Grey A, Scandura G, Guo T, Burke E (2020). Noninvasive Detection of Clinically Significant Prostate Cancer Using Circulating Tumor Cells. J Urol.

[15] SPIRIT 2013 explanation and elaboration: guidance for protocols of clinical trials | The BMJ. Available from: https://www.bmj.com/content/346/bmj.e7586. [cited 2023 May 31]. 10.1136/bmj.e7586 PMC3541470 23303884

[16] Hüsemann Y, Geigl JB, Schubert F, Musiani P, Meyer M, Burghart E (2008). Systemic spread is an early step in breast cancer. Cancer Cell.

[17] Ilie M, Hofman V, Long-Mira E, Selva E, Vignaud JM, Padovani B (2014). ‘Sentinel’ circulating tumor cells allow early diagnosis of lung cancer in patients with chronic obstructive pulmonary disease. PLoS ONE.

[18] Enikeev D, Morozov A, Babaevskaya D, Bazarkin A, Malavaud B (2022). A Systematic Review of Circulating Tumor Cells Clinical Application in Prostate Cancer Diagnosis. Cancers.

[19] Meyer CP, Pantel K, Tennstedt P, Stroelin P, Schlomm T, Heinzer H (2016). Limited prognostic value of preoperative circulating tumor cells for early biochemical recurrence in patients with localized prostate cancer. Urol Oncol.

[20] Liu H, Ding J, Wu Y, Wu D, Qi J (2020). Prospective Study of the Clinical Impact of Epithelial and Mesenchymal Circulating Tumor Cells in Localized Prostate Cancer. Cancer Manag Res.

[21] Davies CR, Guo T, Burke E, Stankiewicz E, Xu L, Mao X, et al. The potential of using circulating tumour cells and their gene expression to predict docetaxel response in metastatic prostate cancer. Front Oncol. 2023 ;12. Available from: https://www.frontiersin.org/articles/10.3389/fonc.2022.1060864 . [cited 2023 May 17]. 10.3389/fonc.2022.1060864 PMC9885040 36727071

[22] Boorjian SA, Thompson RH, Tollefson MK, Rangel LJ, Bergstralh EJ, Blute ML (2011). Long-term risk of clinical progression after biochemical recurrence following radical prostatectomy: the impact of time from surgery to recurrence. Eur Urol.

